# Development of a Physiologically Based Model of Bilirubin Metabolism in Health and Disease and Its Comparison With Real‐World Data

**DOI:** 10.1002/psp4.70183

**Published:** 2026-01-16

**Authors:** Ahenk Zeynep Sayin, Lars Kuepfer

**Affiliations:** ^1^ Institute for Systems Medicine With Focus on Organ Interaction University Hospital RWTH Aachen Aachen Germany; ^2^ Center for Computational Life Sciences, RWTH Aachen University Aachen Germany

**Keywords:** bilirubin, disorders of bilirubin metabolism, interindividual variability, PBPK, population simulation, real‐world data

## Abstract

Bilirubin is a breakdown product of erythrocytes and plays a crucial role in elimination of heme‐containing proteins. After its synthesis in the reticuloendothelial system, unconjugated bilirubin is released into plasma and taken up into the liver. In hepatocytes, bilirubin is conjugated and excreted into the gastrointestinal tract via bile, where it is further converted to urobilinoids. There are various genetic factors causing abnormal bilirubin levels in plasma, such as Gilbert syndrome, Crigler‐Najjar syndrome, Dubin‐Johnson syndrome, and Rotor syndrome. To better understand bilirubin metabolism and its disorders, this study develops a physiologically based computational model incorporating published literature as well as real‐world clinical data from the Explorys database. The model simulates bilirubin levels in both healthy individuals and patients with disorders of bilirubin metabolism. Population simulations show that Gilbert syndrome requires a substantial reduction in UDP‐glucuronosyltransferase 1A1 activity, while Crigler‐Najjar syndrome requires near‐complete loss of its function. In contrast, Dubin‐Johnson syndrome is characterized by a significant impairment of multidrug resistance‐associated protein 2 activity. To also illustrate model behavior under targeted perturbations, we simulated administration of atazanavir in healthy individuals and patients with Gilbert syndrome to investigate its effect on bilirubin levels. Relative to baseline, unconjugated bilirubin maximum concentration (*C*_max_) increased by 34% in healthy individuals but by 67% in Gilbert syndrome. Overall, this study provides a conceptual and mechanistically informed framework for studying bilirubin homeostasis and the functional consequences of drug administration in health and disease.

## Study Highlights

- What is the current knowledge on the topic?
  ○ Bilirubin metabolism involves breakdown of heme into unconjugated bilirubin, which is then conjugated in the liver and excreted into the bile. Disorders such as Gilbert syndrome, Crigler‐Najjar syndrome, Dubin‐Johnson syndrome, and Rotor syndrome disrupt this process, leading to abnormal bilirubin levels.
- What question did this study address?
  ○ We developed a whole‐body physiologically based computational model representing bilirubin metabolism to evaluate the bilirubin levels in health and bilirubin‐related disorders, addressing interindividual variability, disorder‐specific mechanistic alterations, and effects of dynamic perturbations.
- What does this study add to our knowledge?
  ○ The study presents functional analyses of bilirubin homeostasis and specific metabolic disorders. It is the first study to systematically compare simulations of bilirubin metabolism with real‐world clinical data, providing insights into interindividual variability and disorder‐specific patterns.
- How might this change drug discovery, development, and/or therapeutics?
  ○ The model provides a conceptual and mechanistic framework for investigation of drug‐induced perturbations in bilirubin metabolism. The model can in particular be used to explore aberrant states in vulnerable patient subgroups with specific metabolic disorders at the population level.

## 1 Introduction

Bilirubin is produced during heme degradation, mainly from the breakdown of hemoglobin in erythrocytes, with the remaining formed by degradation of hemoproteins. Heme is converted to biliverdin by heme oxygenase, which is then converted to unconjugated bilirubin (UB) by biliverdin reductase [1]. As UB is almost water‐insoluble, it is transported to the liver bound to albumin in the bloodstream [2]. In the liver, bilirubin is conjugated by UDP‐glucuronosyltransferase 1A1 (UGT1A1), which makes bilirubin hydrophilic. Conjugated bilirubin (CB) is then excreted into the bile and secreted into the duodenum. In the gastrointestinal tract, bilirubin is converted into urobilinoids through gut microbial enzymes. Most of the urobilinoids are excreted in the feces, while a smaller portion (~1%) is reabsorbed into the enterohepatic circulation or excreted in urine [3, 4].

Disorders of bilirubin metabolism such as Gilbert syndrome, Crigler‐Najjar syndrome, Dubin‐Johnson syndrome, and Rotor syndrome result from genetic and metabolic conditions leading to abnormal levels of bilirubin [5, 6]. Computational models may support a functional understanding of these disorders, which can significantly affect neurological and overall health status [7].

Existing models of bilirubin metabolism in the literature are largely based on compartmental modeling. For example, Berk et al. studied bilirubin kinetics in normal adults using a three‐compartment model [8]. Similarly, Cobelli et al. focused on a two‐compartment model of bilirubin kinetics in normal, hemolytic, and Gilbert's state [9]. Levitt and Levitt developed a pharmacokinetic model simulating plasma concentrations of both UB and CB in various hyperbilirubinemia pathologies based on the observation that double knockout of the organic anion‐transporting polypeptide 1A (OATP1A) and organic anion‐transporting polypeptide 1B (OATP1B) hepatic transporters in rats increases plasma CB approximately 400‐fold [10]. Yang et al. used a physiologically based pharmacokinetic (PBPK) model, tuned with bilirubin levels from published studies on individuals with disorders of bilirubin metabolism, to distinguish drug‐induced hyperbilirubinemia from liver injury‐related hyperbilirubinemia [11]. Similarly, Dong et al. applied PBPK modeling to assess the relative contributions of UGT1A1 and OATP1B1/3 inhibition by atazanavir in bilirubin elevation [12].

**FIGURE 1:**
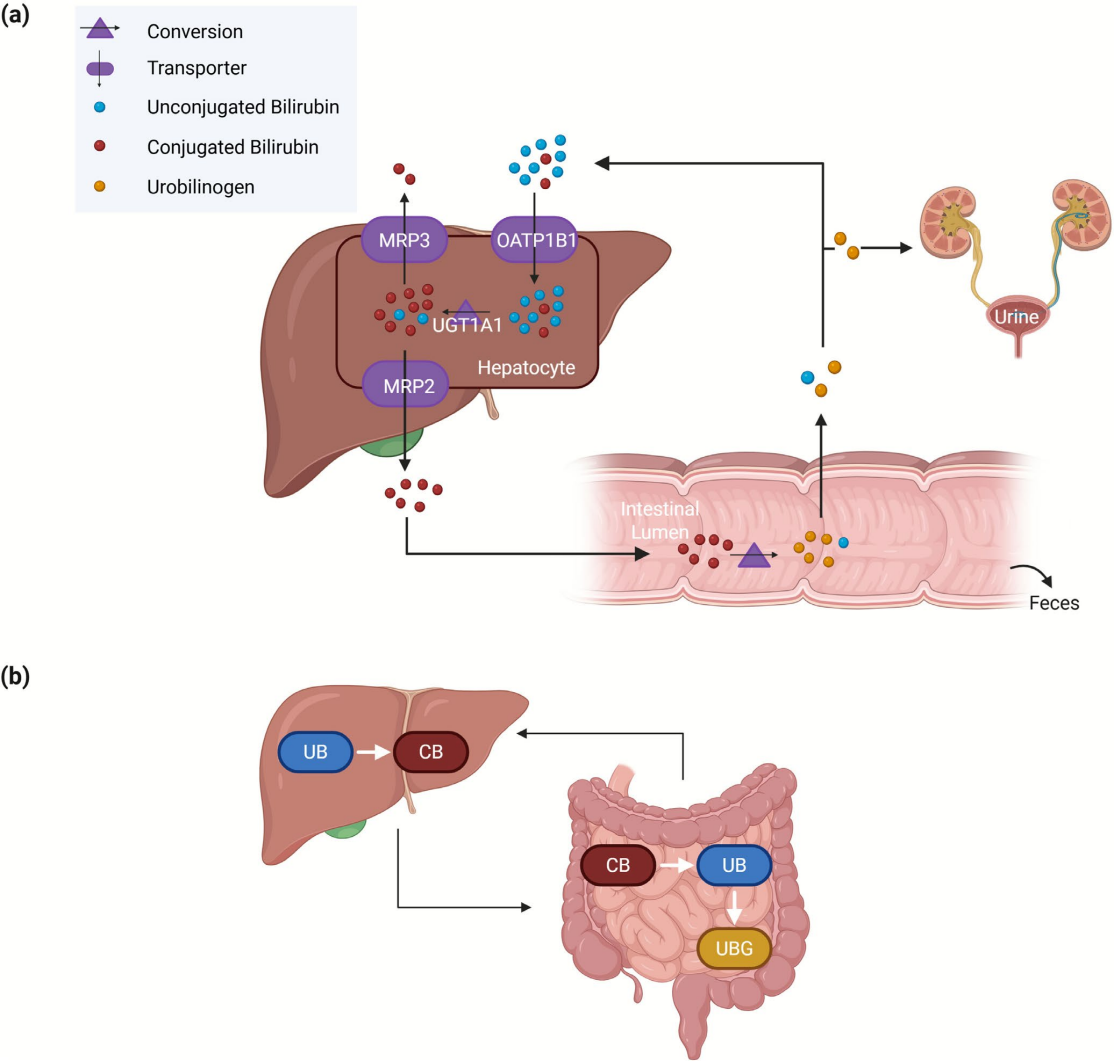
The systemic processes (a) and the specific reactions (b) in the physiologically based computational model of bilirubin metabolism. The model involves bilirubin synthesis, active transport processes via OATP1B1, MRP2, and MRP3, conjugation of bilirubin in the liver, intestinal reduction of bilirubin, as well as fecal and renal excretion. Unconjugated bilirubin (UB) is conjugated in the liver via UGT1A1. After the release of conjugated bilirubin (CB) into the small intestine, conjugated bilirubin is first reduced to unconjugated bilirubin and then further converted to urobilinogen (UBG) via gut microbial enzymes in the intestine. Some unconjugated bilirubin and a small portion of urobilinogen formed in the gut are reabsorbed into the enterohepatic circulation. A minor fraction of urobilinogen enters the systemic circulation, but it is not shown here for simplicity.

We here used PBPK modeling to create a physiologically based computational framework of bilirubin metabolism in health and disease (Figure 1). Generally, PBPK modeling describes physiological processes governing the distribution of drugs in the body at a large level of detail [13]. PBPK modeling can also be used to describe the distribution of endogenous molecules such as bile acids [14, 15] or proteins [16]. The primary goals of the study are to characterize bilirubin levels and to moreover explore the interindividual variability in healthy individuals and bilirubin‐related disorders. To this end, population simulations were performed and compared to published and real‐world data. Extending previous studies using controlled experiments, this work integrates real‐world clinical data from multiple health institutions in the United States, made available by the Explorys database [17]. While model development focused on static conditions, we furthermore explored the potential for dynamic perturbations in bilirubin metabolism. Accordingly, the effect of the UGT1A1 and OATP1B1 inhibitor atazanavir [18] on bilirubin levels was evaluated using models for healthy individuals and patients with Gilbert syndrome. This work hence provides valuable mechanistic insights into the dynamics of bilirubin metabolism in health and disease.

## 2 Methods

### 2.1 Physiologically Based Pharmacokinetic Modeling

Physiologically based pharmacokinetic (PBPK) models predict the absorption, distribution, metabolism, and excretion (ADME) of drugs and endogenous molecules [13]. They rely on extensive physiological information such as organ volumes or organ blood flow rates, usually provided by the PBPK software. Organs are represented by plasma, intracellular, and interstitial compartments interconnected through vascular circulation. In PBPK modeling, tissue permeation and organ/plasma partitioning are calculated from physicochemical properties of a compound [13]. Together with active, protein‐mediated processes, this allows a quantitative simulation of plasma and tissue time‐concentration profiles.

Our model was developed with PK‐Sim and MoBi from the open‐source Open System Pharmacology (OSP) Suite (Version 11.1, www.open‐systems‐pharmacology.org) [19]. Details of the parameter identification and sensitivity analyses are provided in Text S1, and the methods used for calculating partition coefficients and permeabilities are described in Text S2.

### 2.2 Physiologically Based Model of Bilirubin Metabolism

Reactions and active transport processes in the physiologically based model of bilirubin metabolism were modeled using Michaelis–Menten kinetics. Tubular secretion with Michaelis–Menten kinetics was selected for renal excretion, and OATP1B1 and MRP3 was assumed to be exclusively expressed on the basolateral surface of the hepatocytes. To reach the steady state in the model with bilirubin synthesis, simulations were run over an extended period to ensure calibration.

### 2.3 Literature Data

Data points were digitized from published figures using WebPlotDigitizer [20]. Experimental studies involving administration of labeled bilirubin and time‐resolved measurements were additionally considered for model development. In the corresponding simulation, only intravenous administration of 0.5 mg of exogenous bilirubin [21] was simulated, neglecting endogenous bilirubin metabolism.

### 2.4 Explorys Data

The Explorys database is a commercial database comprising electronic health records collected from health institutions in the United States involving data of almost 1 million patients with liver‐related health conditions [17]. Data was retrieved from the Explorys database in September 2024.

Details on cohort selection, exclusion criteria, and bilirubin measurement extraction from the Explorys database are provided in Text S3. Descriptive information for each cohort, including number of patients, age, number of bilirubin observations, and summary statistics are provided in Section 3. All bilirubin measurements with the dimension of mass/volume were harmonized to mg/dL units. Statistics were calculated by excluding the top and bottom 2.5% of the data as there were some extreme outliers.

### 2.5 Population Simulations

For the population simulations, the model was imported from MoBi into PK‐Sim and a virtual population of 1000 individuals was created. PK‐Sim randomly varies anatomical and physiological parameters according to their respective distributions [22]. To account for biological variability, a log‐normal distribution of *V*_max_ values was applied, with a geometric standard deviation of 1.6, based on Beal's characterization of typical genetic expressions variability [23]. For disease‐specific cohorts, *V*_max_ of the affected enzyme or transporter was scaled to reproduce the deficiency in each disorder. This top‐down *V*_max_ scaling approach was chosen to reflect alterations observed in real‐world data while maintaining simplicity and computational efficiency. Additionally, the bottom and top 2.5% of the data were trimmed to ensure comparability with the Explorys data.

### 2.6 Simulation of Atazanavir Administration

The atazanavir PBPK model from the OSP Suite PBPK Model library was used [24]. It includes metabolism by CYP3A4, mechanism‐based inhibition of CYP3A4, mixed inhibition of UGT1A1, and glomerular filtration. Moreover, competitive inhibition of OATP1B1 by atazanavir (*K*_i_ = 0.129 μM) was incorporated [12]. Combined with the bilirubin metabolism model, 400 mg once daily oral administration of atazanavir for 30 days has been simulated in healthy individuals and patients with Gilbert syndrome.

## 3 Results

In this study, we developed a physiologically based computational model of bilirubin metabolism, involving key enzymatic and transport processes. The study aimed to evaluate the bilirubin levels in health and bilirubin‐related disorders, and to address interindividual variability using published and real‐world data. Published data was used as the primary reference in the model calibration, while real‐world data served as a second level of comparison to assess population‐level variability. Furthermore, we also investigated the effect of the UGT1A1 and OATP1B1 inhibitor atazanavir in healthy individuals and patients with Gilbert syndrome.

### 3.1 Model for Healthy Individuals and Rotor Syndrome

The physiologically based model includes bilirubin synthesis, hepatic uptake of both UB and CB by OATP1B1 [25, 26, 27], and bilirubin conjugation via UGT1A1 (Figure 1). Competitive inhibition between UB and CB for OATP1B1 was considered to capture the increase in UB levels in CB pathologies [10] and the *K*_i_ values for the competing species were assumed to be equal to their *K*_m_ for OATP1B1. The model also involves multidrug resistance‐associated protein 2 (MRP2) mediated biliary excretion, multidrug resistance‐associated protein 3 (MRP3) mediated systemic efflux, and reduction of CB to UB and further to urobilinogen via gut microbial enzymes. Additionally, the model accounts for fecal and urinary excretion, as well as enterohepatic circulation. An alternative OATP1B1‐independent hepatic uptake of UB [10], represented as a first‐order process, was also included to account for basal uptake activity in patients with Rotor syndrome. Passive transport is explicitly represented in the underlying PBPK model. The high affinity of UB for albumin is represented in the model by an extremely low fraction unbound [28] (Table S1). Synthesis of UB was assumed to be 260 mg/day [21], modeled as a constant flux in venous plasma.

**FIGURE 2:**
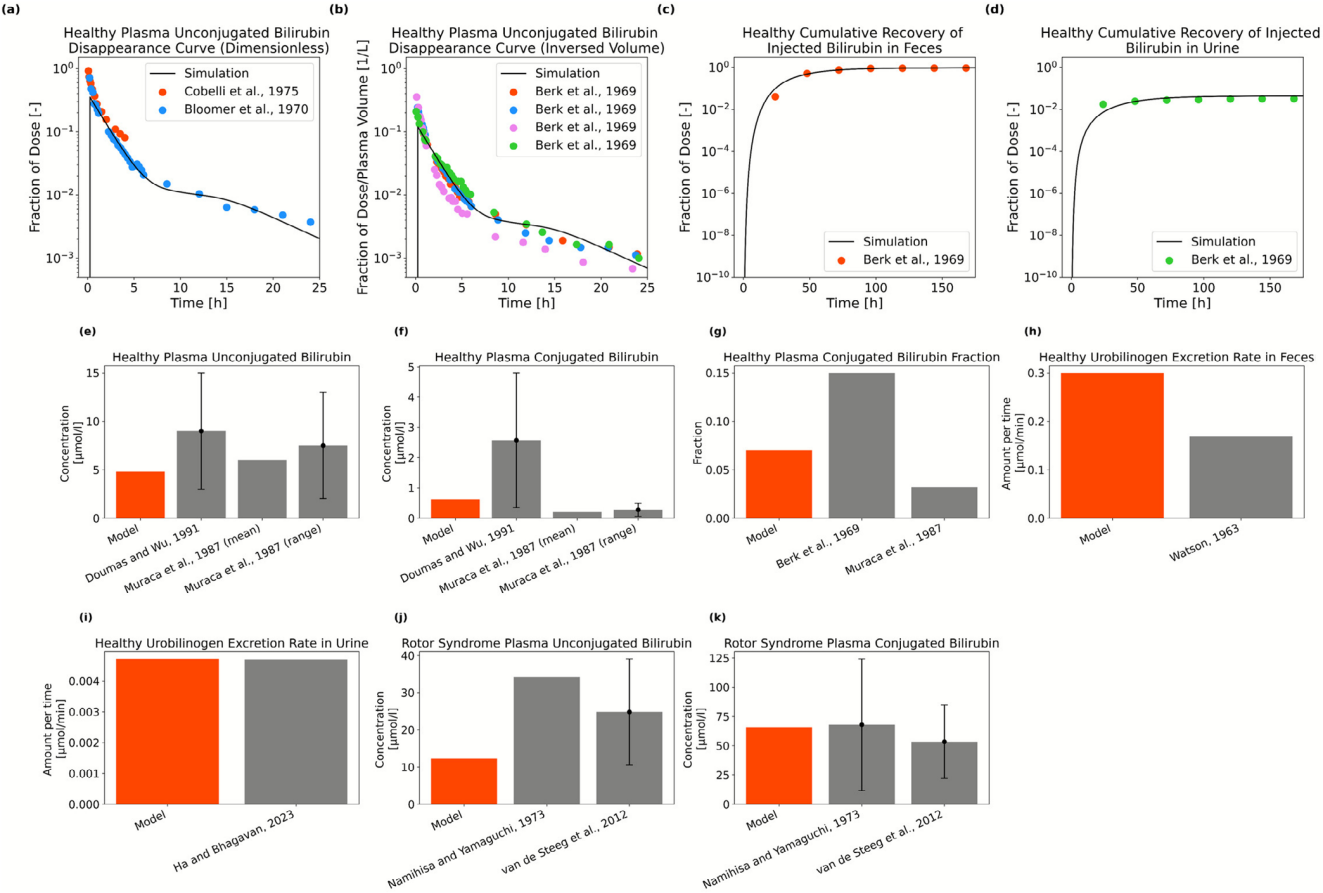
Model predictions of the bilirubin metabolism model. Panels (a, b) show the predicted healthy plasma unconjugated bilirubin disappearance curves following bilirubin administration, presented as dimensionless (a) and per plasma volume (b), along with the measurements from Cobelli et al. [9], Bloomer et al. [21] and Berk et al. [8]. Panels (c, d) present simulated healthy cumulative recovery of injected bilirubin in feces and urine, respectively, in comparison to the measurements from Berk et al. [8]. Panels (e, f) show the healthy plasma unconjugated and conjugated bilirubin concentrations, respectively, with the measurements from Doumas and Wu [29] and Muraca et al. [30] plotted alongside model predictions at the steady state. For the data with error bars in these panels, the bars represent the upper and lower bounds of the range, while the height of the bar indicates the midpoint of this range. Panel (g) shows the model prediction of the healthy plasma conjugated bilirubin fraction, compared with the results from Berk et al. [8] and Muraca et al. [30]. Panels (h, i) display healthy fecal and urinary urobilinogen excretion rates, respectively, with the model outputs compared to data from Watson [31] and Ha and Bhagavan [32]. Panels (j, k) represent model outputs for plasma unconjugated and conjugated bilirubin concentrations in Rotor syndrome, respectively, in comparison to data from Namihisa and Yamaguchi [33] and van de Steeg et al. [34] with the error bars showing standard deviation.

For the model development, intravenous administration of exogenous bilirubin was initially considered to describe time‐resolved experimental data with labeled bilirubin studies (Figure 2a–d) [8, 9, 21]. Kinetic parameters in Table S2 were calibrated using published data from both healthy individuals and patients with Rotor syndrome (Table S3), following an approach similar to that of Levitt and Levitt [10], but additionally incorporating time‐dependent data from labeled bilirubin studies by Cobelli et al. [9], Bloomer et al. [21], and Berk et al. [8] for healthy individuals. Data from healthy individuals and Rotor syndrome were used simultaneously in the calibration to inform the rate of OATP‐independent hepatic uptake. In the model for Rotor syndrome, OATP1B1‐mediated transport was excluded, while all other transport pathways were present. For steady state predictions (Figure 2e–k), the model including bilirubin synthesis was used, with absent OATP1B1 activity for Rotor syndrome.

In healthy state, the model could successfully describe the plasma levels of UB and CB (Figure 2a,b,e,f), including the CB fraction within the range reported in literature (Figure 2g). Moreover, the model reproduces the cumulative recovery of injected bilirubin in feces and urine from labeled bilirubin studies (Figure 2c,d). However, it overestimates the rate of fecal excretion of urobilinogen (Figure 2h), although the predicted rate of urinary excretion aligns with the literature (Figure 2i). The average rates reported in the literature range from 64 mg/day [21, 35] to 173 mg/day [21, 36] with individual excretion rates of 40.9–279.4 mg/day [35]. Our model estimates a fecal excretion rate of 255 mg/day, which remains within plausible limits.

Sensitivity analysis was performed on the healthy individual model to assess the effect of identified kinetic parameters on the model output. Although a formal identifiability analysis was not conducted due to computational cost, the sensitivity results provide insight into parameter identifiability. Parameters with high sensitivities are expected to be more identifiable as their variation strongly affects outputs. The parameter with the highest sensitivity is OATP1B1 *V*_max_ of CB, followed by OATP1B1 *K*_m_ and MRP2 *V*_max_ of CB. In contrast, the renal excretion rate of urobilinogen shows the lowest sensitivity (Table S4).

In Rotor syndrome, the model captures the characteristic pattern, replicating the limited increase in UB (2.55‐fold) while showing a higher elevation in CB (106.2‐fold), with the simulated UB and CB concentrations being within the observed range (Figure 2j,k) [33, 34].

To also account for the interindividual variability in bilirubin metabolism, simulations were performed with a virtual population of 1000 individuals, which also serves as a validation of the model considering the range of plasma bilirubin levels.

Simulated population‐level bilirubin levels in healthy individuals and Rotor syndrome were compared with the Explorys data and literature (see Section 2). To define a healthy bilirubin metabolism cohort in the Explorys database, the cases with ICD‐10 codes affecting bilirubin metabolism were excluded (see Text S3) and individuals aged 18–65 were considered in all cohorts (Table 1).

**TABLE 1:** Statistics of bilirubin observations in the Explorys database for the healthy bilirubin metabolism cohort and cohorts with disorders of bilirubin metabolism.

		Total bilirubin	Unconjugated bilirubin	Conjugated bilirubin
Healthy	Number of patients	224,827	23,744	73,132
	Age (years), mean ± SD	52.61 ± 10.10	51.82 ± 10.23	52.60 ± 9.95
	Age (years), range	18–65	18–65	18–65
	Number of female patients, (%)	127,323 (56.6%)	13,163 (55.4%)	40,548 (55.4%)
	Number of observations	2,469,047	65,979	265,663
	Observation, mean ± SD (mg/dL)	0.58 ± 0.40	0.43 ± 0.34	0.37 ± 0.46
Gilbert syndrome	Number of patients	346	63	178
	Age (years), mean ± SD	48.40 ± 12.25	46.62 ± 12.03	47.53 ± 12.87
	Age (years), range	18–65	18–65	18–65
	Number of female patients, (%)	83 (24.0%)	12 (19.0%)	38 (21.3%)
	Number of observations	931	81	260
	Observation, mean ± SD (mg/dL)	2.82 ± 2.45	1.79 ± 0.81	0.80 ± 0.75
Crigler‐Najjar syndrome	Number of patients	1	/	/
	Age (years), mean ± SD	30.00 ± 0.00	/	/
	Age (years), range	/	/	/
	Number of female patients, (%)	0 (0.00%)	/	/
	Number of observations	2	/	/
	Observation, mean ± SD (mg/dL)	4.8 ± 0.00	/	/
Dubin‐Johnson and Rotor syndromes	Number of patients	7152	1280	3517
	Age (years), mean ± SD	49.22 ± 11.94	47.69 ± 12.29	48.01 ± 12.40
	Age (years), range	18–65	18–65	18–65
	Number of female patients, (%)	2825 (39.5%)	540 (42.2%)	1,1417 (40.3%)
	Number of observations	52,761	2762	8819
	Observation, mean ± SD (mg/dL)	8.02 ± 7.84	2.61 ± 2.06	5.66 ± 5.52

**FIGURE 3:**
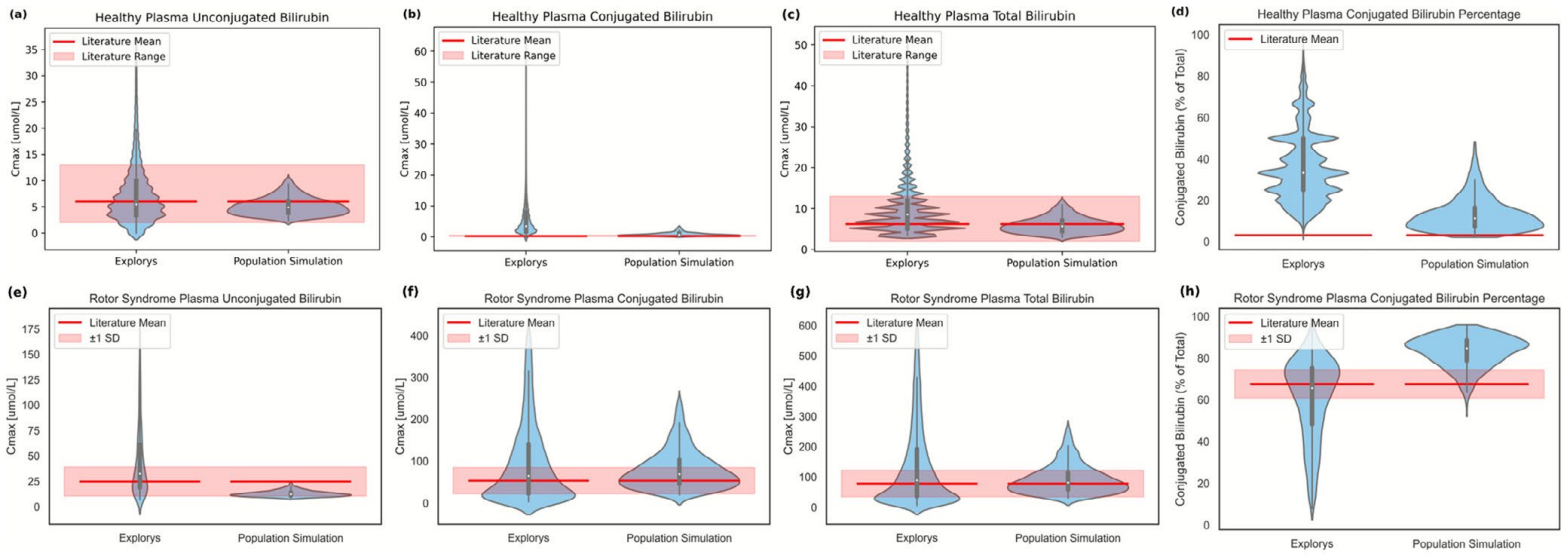
Plasma unconjugated, conjugated, total bilirubin levels, and conjugated bilirubin ratio of population simulations in comparison to the literature and the Explorys data for the cohort of healthy bilirubin metabolism (a–d) and Rotor syndrome (e–h). These simulations were performed using the calibrated model without further parameter adjustment and therefore represent independent validation. For the healthy state, the literature means, ranges, and conjugated bilirubin ratio are reported in Muraca et al. [30]. For the Rotor syndrome, the literature means, ranges, and conjugated bilirubin ratio are reported in van de Steeg et al. [34].

In healthy individuals, the median UB and total bilirubin (TB) levels from Explorys and the population simulation align with the literature means (Figure 3a–d). For both UB and TB, the range of Explorys is wider, though most individuals fall within the literature‐reported ranges, whereas the population simulation results are completely within literature ranges. For the plasma CB, Explorys has higher concentrations than the literature‐reported range, while the population simulation results fall mostly within the literature range. For the healthy CB ratio, Explorys and population simulation results generally exhibit higher values than literature, with the population simulation having the highest density slightly above but closer to the literature mean, while covering most of the range observed in the Explorys data. Sensitivity analysis of the rate constants indicates that hepatic uptake of CB and UB via OATP1B1 has the greatest impact on their plasma levels in healthy individuals (Table S4).

In Rotor syndrome, the Explorys data exhibit broad distribution; however, median UB, CB, and TB concentrations, as well as the CB ratio, align with the literature‐reported means (Figure 3e–h). Population simulation predicts median CB and TB concentrations matching literature‐reported means, while UB concentrations are lower but remain within the literature range. Some simulated CB ratios match the literature, although most simulation results cluster slightly at a higher range. In Rotor syndrome, the observed CB ratio is typically above 50% [33]. Based on sensitivity analysis of rate constants (Table S4), MRP2 and OATP1B1‐independent pathways primarily affect CB and UB plasma levels in Rotor syndrome, respectively, suggesting that impaired hepatic uptake makes bilirubin disposition more dependent on passive diffusion and biliary excretion by MRP2.

### 3.2 Other Disorders of Bilirubin Metabolism

Next, we investigated the functional consequences for various disorders of bilirubin metabolism, focusing on Gilbert syndrome, Crigler‐Najjar syndrome, and Dubin‐Johnson syndrome. In Gilbert syndrome and Crigler‐Najjar syndrome, UGT1A1 activity is decreased, whereas MRP2 activity is reduced in Dubin‐Johnson syndrome [5, 6]. A population simulation of 1000 individuals was performed, recalibrating only the mean *V*_max_ of the affected enzyme or transporter for each disorder, focusing on the most affected bilirubin species, that is, UB for Gilbert syndrome and Crigler‐Najjar syndrome, and CB for Dubin‐Johnson syndrome. A geometric standard deviation of 1.6 [23] was applied to all *V*_max_. The results of population simulations for other disorders of bilirubin metabolism were compared to the bilirubin levels reported in the literature (Table S5) and Explorys [10, 30, 37].

**FIGURE 4:**
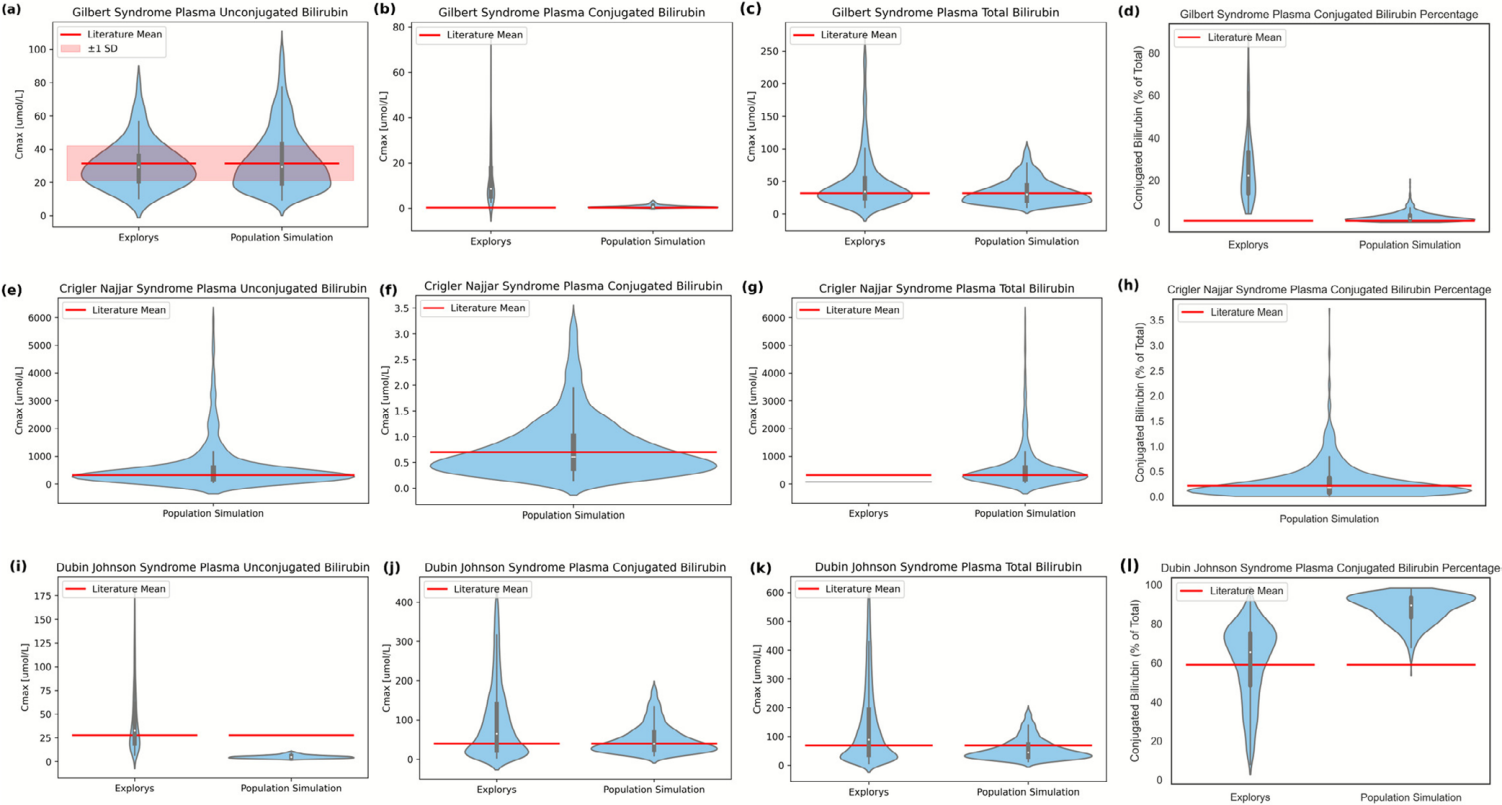
Plasma unconjugated, conjugated, total bilirubin levels, and conjugated bilirubin ratio of population simulations in comparison to the literature and the Explorys data for other disorders of bilirubin metabolism. Panels (a–d), (e–h), and (i–l) show the plasma bilirubin levels and conjugated bilirubin ratio in Gilbert syndrome, Crigler‐Najjar syndrome, and Dubin‐Johnson syndrome, respectively. For each disorder, only the *V*_max_ of the affected enzyme or transporter was recalibrated to reproduce the levels of the most affected bilirubin species in each disorder (unconjugated bilirubin for Gilbert and Crigler‐Najjar syndromes, conjugated bilirubin for Dubin‐Johnson syndrome). The literature values are reported in Table S5 [10, 30, 37].

For Gilbert syndrome, the mean *V*_max_ of UGT1A1 was set to 4.60 μmol/L/min, which is 0.75% of the value in the healthy‐state reference model. Literature estimates UGT1A1 activity in Gilbert syndrome as 30%–50% of normal [38, 39]. Crigler‐Najjar Type 2 is associated with UGT1A1 activity around 10% of the healthy reference [6, 39]. Crigler‐Najjar Type 1, a more severe disorder, results from complete or near absence of UGT1A1. In our model, the mean *V*_max_ of UGT1A1 for Crigler‐Najjar syndrome was reduced to 0.55 μmol/L/min (0.09% of healthy‐state value), which is consistent with literature [6]. In Dubin‐Johnson syndrome, conjugated hyperbilirubinemia results from the absent or minimal MRP2 activity [5, 6]. For the Dubin‐Johnson syndrome, mean *V*_max_ of MRP2 was decreased to 2.53 μmol/L/min, representing 1.5% of the healthy‐state value (Figure 4).

In Gilbert syndrome, the mean values in literature are reflected by the medians of the population simulation for UB, CB, and TB concentrations, as well as CB ratio (Figure 4a–d). In Explorys data, UB and TB align with the literature mean, while CB concentrations and ratios are higher than literature values. For UB, most of the concentrations from both Explorys and the population simulation remain within the literature‐reported variation.

In Crigler‐Najjar syndrome, the literature mean is well represented by the median of population simulation for all bilirubin concentrations and CB ratio (Figure 4e–h). The population simulations show a broad range of UB and TB levels, although Explorys data are limited to two TB measurements from the same patient, both below the literature mean. Sensitivity analysis of rate constants (Table S4) shows that, in Gilbert and Crigler‐Najjar syndromes, UB plasma levels are primarily influenced by UGT1A1, whereas CB levels are mostly affected by OATP1B1‐mediated uptake. This indicates that under decreased UGT1A1 activity, the reduced conjugation rate is the major driver of UB accumulation, while OATP1B1 is the main pathway regulating CB levels.

For Dubin‐Johnson syndrome, TB medians from both Explorys data and population simulations align with the literature mean (Figure 4i–l). The median of population simulation for CB matches the literature mean, whereas the median of the Explorys data is slightly higher. For UB, Explorys data and the literature mean are similar, while the simulation results are lower but still fall within the range of the Explorys data. The CB ratio is higher than the literature‐reported mean for both Explorys data and the population simulation, while their peaks of the distributions are comparable. Sensitivity analysis of rate constants (Table S4) indicates that OATP1B1 pathway primarily affects plasma UB and CB levels in Dubin‐Johnson syndrome, suggesting that impaired biliary excretion results in UB and CB levels being mainly influenced by hepatic uptake.

Overall, compared to real‐world clinical data, population simulations reflect the central and most common values in clinical data, while real‐world data generally show larger variability.

### 3.3 Subject‐Specific Bilirubin Profiles of Populations

To better understand individual‐level bilirubin levels of these various populations, we examined absolute plasma concentrations of UB and CB for both real‐world and simulated patients, underlying the ratios shown in Figures 3 and 4. This allows us to assess correlations between real‐world UB and CB using linear regression in different states and to compare observed values with simulations.

**FIGURE 5:**
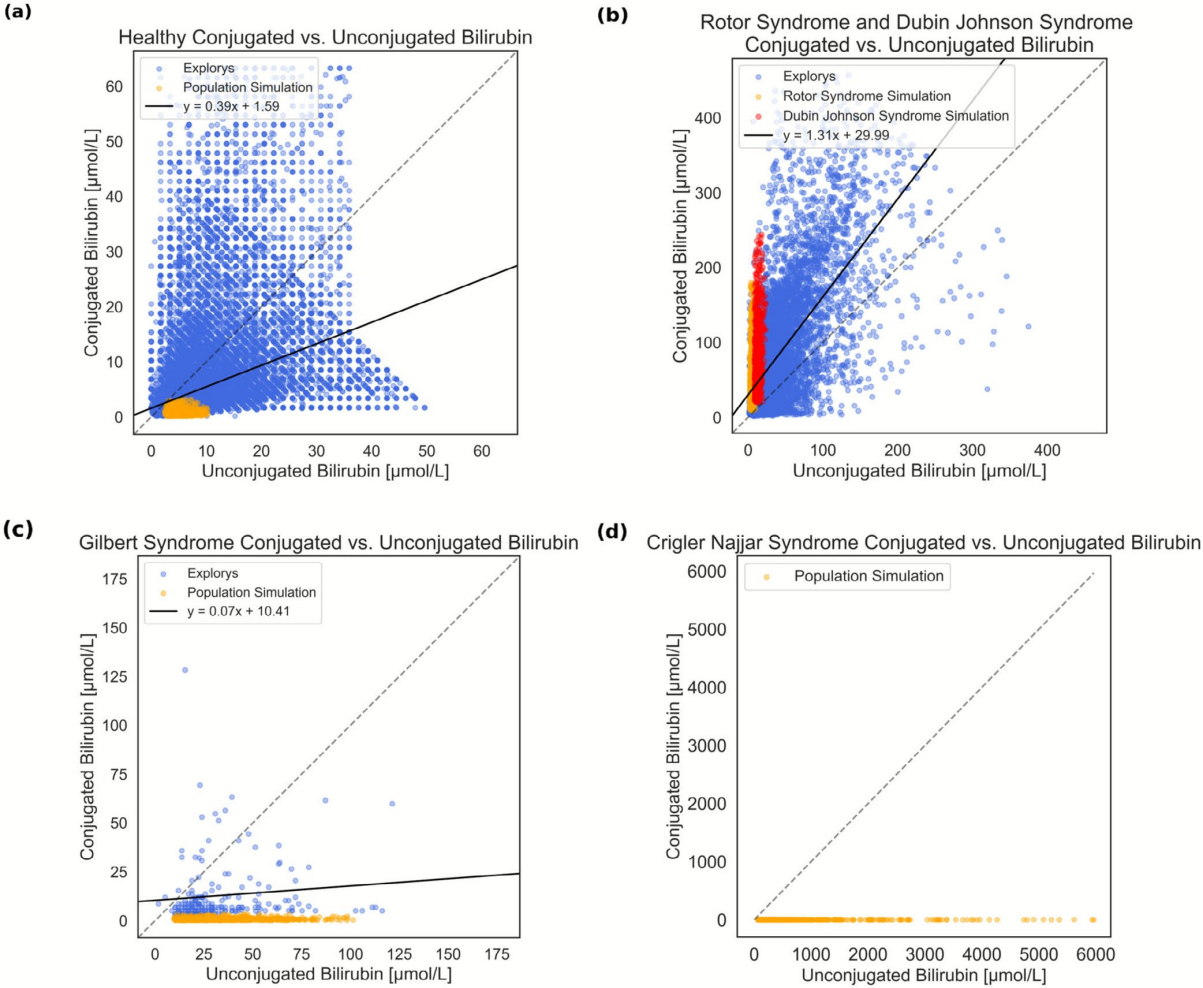
Conjugated versus unconjugated bilirubin concentrations for individual patients. Panels (a–c) show both conjugated and unconjugated bilirubin levels of individuals from real‐world clinical data (blue) and from simulations (yellow and red) representing healthy individuals, Rotor and Dubin‐Johnson syndromes, and Gilbert syndrome, respectively. Rotor and Dubin‐Johnson syndromes are shown in the same panel as they share the same ICD‐10 code in the Explorys database. Panel (d) shows only the simulated bilirubin levels of patients with Crigler‐Najjar syndrome since there is no Explorys data available. Dashed line represents the identity line, and the solid line represents the linear regression of real‐world clinical data, with the corresponding equation provided in the legend.

Figure 5 compares simulated and real‐world CB versus UB levels under different conditions. In healthy individuals, CB and UB levels show a moderate positive correlation (*y* = 0.39*x* + 1.59), where small increases in UB are accompanied by a proportional rise in CB. In Rotor and Dubin‐Johnson syndromes, both bilirubin species are elevated, with a stronger correlation (*y* = 1.31*x* + 29.99), reflecting greater elevation of CB relative to UB. In Gilbert syndrome, UB levels are elevated, while CB remains low and weakly correlated (*y* = 0.07*x* + 10.41). The simulated individuals match the high‐density regions of the real‐world data although they do not capture the full observed variability.

### 3.4 Atazanavir Administration in Healthy Individuals and Gilbert Syndrome

**FIGURE 6:**
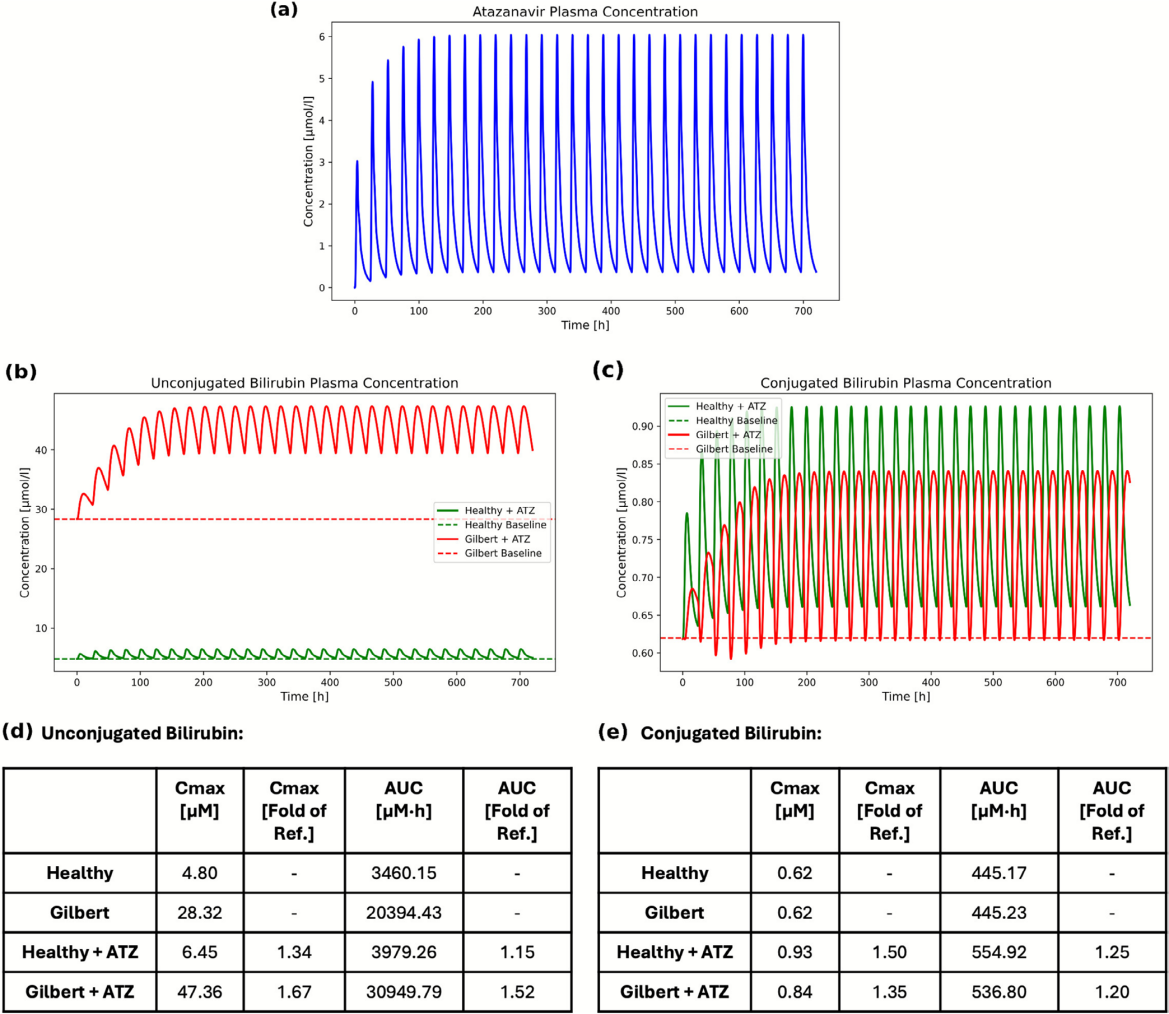
Simulated plasma bilirubin levels following atazanavir administration in a healthy individual and an individual with Gilbert syndrome. Panel (a) shows the simulated plasma atazanavir concentration. Panels (b, c) show the simulated plasma unconjugated and conjugated bilirubin levels in a healthy individual and an individual with Gilbert syndrome. Dashed lines indicate the bilirubin concentrations prior to atazanavir administration. Panels (d, e) show the maximum concentration (*C*_max_) and area under the curve (AUC) in both individuals, presented in absolute units and as fold‐change relative to each individual's baseline model.

So far, only static cases have been considered with the physiologically based model. To illustrate its application for dynamic system‐level perturbations, we simulated the time‐dependent effect of atazanavir administration on time‐concentration profiles of various bilirubin species. Atazanavir is an antiviral protease inhibitor used in the treatment of HIV [40]. It also inhibits UGT1A1 and OATP1B1, leading to hyperbilirubinemia in some patients [41]. Gilbert syndrome has been suggested as a potential risk factor for atazanavir‐induced hyperbilirubinemia [41]. We here simulated the effect of atazanavir on a healthy individual compared to someone with Gilbert syndrome. The impact of 30‐day atazanavir administration on plasma bilirubin levels in health and Gilbert syndrome is presented (Figure 6).

The results show that the atazanavir plasma concentration reached steady state within 30 days (Figure 6). In healthy individuals, UB *C*_max_ increased by 34% and AUC by 15%, while remaining within the normal range [29]. In Gilbert syndrome, atazanavir caused a greater response in UB, increasing *C*_max_ by 67% and AUC by 52%, while baseline UB levels were already above the normal range. In healthy individuals, CB *C*_max_ and AUC rose by 50% and 25%, whereas individuals with Gilbert syndrome showed an elevation of 35% and 20%, respectively. In both individuals, CB was within the normal range [29]. Overall, the results indicate that atazanavir has a greater impact in Gilbert syndrome, potentially posing a clinical risk.

## 4 Discussion

This study developed a physiologically based computational model simulating bilirubin metabolism in both healthy individuals and patients with disorders of bilirubin metabolism. The model involves key processes, including hepatic uptake of bilirubin, its conjugation, and its biliary excretion. It accurately replicated healthy plasma bilirubin levels and urinary excretion. However, the fecal excretion rate was overestimated. This discrepancy may reflect the variability in fecal excretion rates, affected by diet or bowel habits [35]. For Rotor syndrome, the plasma bilirubin levels with the typical pattern of limited increase in UB and a larger elevation in CB were reproduced.

Besides individual simulations of healthy reference and Rotor syndrome, a virtual population of 1000 individuals was simulated. Incorporating real‐world clinical data, the study also reveals alterations in various disorders and allows comparisons with controlled‐study values, showing less variability than clinical data. Moreover, our dynamic model enabled simulation of atazanavir‐induced bilirubin changes, demonstrating its potential relevance for assessing risk in individuals with impaired bilirubin metabolism. For the disease‐specific virtual populations, *V*_max_ of the affected enzyme/transporter was scaled to reproduce characteristic bilirubin profiles. This top‐down *V*_max_ scaling is appropriate because each disorder is predominantly driven by a single enzymatic or transporter defect altering bilirubin kinetics [5, 6, 34, 42].

Comparison with the model of Levitt and Levitt [10] shows both consistencies and differences in bilirubin predictions across different conditions (Table S6). Our model is a whole‐body dynamic framework that can simulate dynamic perturbations such as drug‐induced hyperbilirubinemia, whereas Levitt and Levitt's model is a steady‐state framework focused primarily on clinical bilirubin measurements. Both models capture baseline bilirubin in healthy individuals, with our population simulation yielding a higher median for CB. In disorders, both models reproduce expected trends, but in some cases differ quantitatively.

In a previous publication by Dong et al. [12] hyperbilirubinemia was analyzed as a result of UGT1A1 and OATP1B1/3 inhibition by atazanavir. For their study, the authors used a PBPK model which represented bilirubin synthesis through intravenous infusions. In this earlier work, different UGT1A1 phenotypes (extensive, intermediate and poor metabolizers, respectively) were considered instead of disease‐specific populations. The different UGT1A1 metabolizer phenotypes in the publication by Dong et al. have been identified from three specific total bilirubin baseline levels. Also, the liver was modeled as a permeability‐limited tissue and the published renal clearance rate for CB as well as in vitro UGT1A1 kinetics for UB were used as inputs for the elimination [12]. In contrast, our model accounts for both passive and active hepatic uptake, which is consistent with evidence for combined OATP1B1/3‐mediated and passive transport [43, 44]. Moreover, in our model we assumed competitive inhibition between UB and CB for OATP1B1, supported by a rise in UB observed in CB pathologies [10]. Interestingly, the simulations in the publication by Dong et al. show larger effects on total bilirubin as well as an increase in unconjugated bilirubin after administration of atazanavir [12]. A major difference to the previous work is that in our model UGT1A1 activity in Gilbert syndrome only represents 0.75% of the healthy reference. Since the UGT1A1 phenotypes in Dong et al. have a much larger activity, this could explain the lower sensitivity of our model towards atazanavir‐mediated inhibition.

Our model describes the various syndromes of bilirubin metabolism mechanistically. Furthermore, our work is the first study on modeling of bilirubin metabolism to systematically compare simulation results with real‐world data. Moreover, this is also the first whole‐body model capable of describing time‐resolved experimental data with labeled bilirubin.

Despite reasonably representing the bilirubin metabolism across different disorders, the model has some limitations. Most urobilinogen is oxidized to stercobilin for fecal excretion, with a smaller fraction forming urobilin excreted in urine. To avoid unnecessary model complexity, stercobilin and urobilin are represented as urobilinogen, with adjusted lipophilicity and solubility to match reported excretion rate. Additionally, data comparability across different institutions in the Explorys database presents another challenge. Although real‐world data provides insights into the population‐level variability, it may not always align with controlled clinical studies due to heterogeneity in data sources. Differences in protocols, lab methods, and recording standards may introduce inconsistencies in bilirubin data. Lastly, ICD‐10 codes were used to identify the disorders of bilirubin metabolism. However, Rotor syndrome and Dubin‐Johnson syndrome share the same code. While the cohort may include both, given the rarity of Rotor syndrome (0.0001% prevalence) [45], the cohort likely consists mainly of individuals with Dubin‐Johnson syndrome.

Future work could extend the model to liver diseases like cirrhosis or cholestasis to study effects on bilirubin kinetics. Additionally, the model could also assess risks of complications like kernicterus in various pathological states.

In conclusion, this study presents a physiologically based computational model simulating bilirubin metabolism in healthy individuals and patients with bilirubin metabolism disorders. Disorders are represented by adjusting parameters from the healthy reference model. Using clinical and literature data, the model allows functional analyses across different conditions, despite some deviations in fecal excretion rates in the healthy state. Furthermore, its dynamic nature enables simulation of drug effects on bilirubin levels, allowing potential assessment of hyperbilirubinemia risk in high‐risk cohorts under various conditions.

## Author Contributions

A.Z.S. and L.K. wrote the manuscript and designed the research. A.Z.S. performed the research and analyzed the data.

## Funding

This study was funded by the German Federal Ministry of Research, Technology and Space (BMFTR), grant number 03LW0304K.

## Conflicts of Interest

The authors declare no conflicts of interest.

## Supporting information


**Data S1:** psp470183‐sup‐0001‐DataS1.zip.
