# Supplementary material for: Development of a Physiologically Based Model of Bilirubin Metabolism in Health and Disease and Its Comparison With Real‐World Data
Source: CPT Pharmacometrics Syst Pharmacol. 2026 Jan 16;15(2):e70183. doi: 10.1002/psp4.70183 (PMC12823313; doi:10.1002/psp4.70183)
Supplement: Supplementary file 1 — Data S1: psp470183‐sup‐0001‐DataS1.zip. [file PSP4-15-e70183-s001.zip › psp470183-sup-0001-Supinfo.docx]

**Table S1.** Physicochemical properties of bilirubin species used in the model.

|  | **Property** | **Value** | **Reference** |
| --- | --- | --- | --- |
| Bilirubin (Unconjugated Bilirubin) | MW [g/mol] | 584.66 | [1] |
|  | Solubility [g/l] | 9.60E-3 | [1] |
|  | logP [Log Units] | 3.22 | [1] |
|  | FU [-] | 2.70E-5 | [2] |
|  | Cellular Partition Coefficient Method | PK-Sim Standard | [3] |
|  | Cellular Permeability | PK-Sim Standard | [3] |
|  | Interstitial Partition Coefficient Method | Schmitt | [3] |
|  | Intestinal Permeability Calculation Method | PKSim | [3] |
| Bilirubin diglucuronide  (Conjugated Bilirubin) | MW [g/mol] | 936.92 | [1] |
|  | Solubility [g/l] | 0.13 | [1] |
|  | logP [Log Units] | 1.32 | [1] |
|  | FU [-] | 0.02 | [2] |
|  | Cellular Partition Coefficient Method | PK-Sim Standard | [3] |
|  | Cellular Permeability | PK-Sim Standard | [3] |
|  | Interstitial Partition Coefficient Method | Schmitt | [3] |
|  | Intestinal Permeability Calculation Method | PKSim | [3] |
| Urobilinogen | MW [g/mol] | 590.71 | [1] |
|  | Solubility [g/l] | 13.89 | Fitted parameter |
|  | logP [Log Units] | 1.44 | Fitted parameter |
|  | FU [-] | 0.2 | [4] |
|  | Cellular Partition Coefficient Method | PK-Sim Standard | [3] |
|  | Cellular Permeability | PK-Sim Standard | [3] |
|  | Interstitial Partition Coefficient Method | Schmitt | [3] |
|  | Intestinal Permeability Calculation Method | PKSim | [3] |

**Table S2.** Fitted kinetic parameters.

| **Reaction/Process** | **Parameter** | **Value** |
| --- | --- | --- |
| Urobilinogen-Renal clearances | Km [µmol/l] | 1.14E-3 |
| Urobilinogen-Renal clearances | TSmax [µmol/l/min] | 1.33 |
| cBilirubin-MRP2 | Km [µmol/l] | 144.85 |
| cBilirubin-MRP2 | Vmax [µmol/l/min] | 168.47 |
| Urobilinogen-MRP2 | Km [µmol/l] | 2486.55 |
| Urobilinogen-MRP2 | Vmax [µmol/l/min] | 1233.39 |
| cBilirubin-MRP3 | Km [µmol/l] | 69.25 |
| cBilirubin-MRP3 | Vmax [µmol/l/min] | 25.40 |
| uBilirubin-OATP1B1 | Km [µmol/l] | 4.62 |
| uBilirubin-OATP1B1 | Vmax [µmol/l/min] | 3580.49 |
| cBilirubin-OATP1B1 | Km [µmol/l] | 98.05 |
| cBilirubin-OATP1B1 | Vmax [µmol/l/min] | 192.06 |
| Urobilinogen-OATP1B1 | Km [µmol/l] | 5.09 |
| Urobilinogen-OATP1B1 | Vmax [µmol/l/min] | 11.01 |
| uBilirubin-UGT-cBilirubin | Km [µmol/l] | 0.62 |
| uBilirubin-UGT-cBilirubin | Vmax [µmol/l/min] | 612.98 |
| cBilirubin-GUS-uBilirubin | Km [µmol/l] | 22.07 |
| cBilirubin-GUS-uBilirubin | Vmax [µmol/l/min] | 312651.42 |
| uBilirubin-BilR-Urobilinogen | Km [µmol/l] | 809.71 |
| uBilirubin-BilR-Urobilinogen | Vmax [µmol/l/min] | 4.50 |
| uBilirubin_Synth | Rate of reaction [µmol/min] | 0.31 |
| Alternative uBilirubin uptake path | Rate constant [1/min] | 406.59 |

**Table S3.** Bilirubin plasma levels in healthy individuals and Rotor syndrome.

|  | **Measurement** | **Value** | **Unit** | **Number of Subjects** | **Used in Model Calibration** | **Reference** |
| --- | --- | --- | --- | --- | --- | --- |
| Healthy | Unconjugated bilirubin in plasma | 6.0 | μM | 32 | Yes | [5, 6] |
|  |  | 2.0-13 | μM | 32 | Yes | [5, 6] |
|  |  | 3-15 | μM | 109 | Yes | [6, 7] |
|  | Conjugated bilirubin in plasma | 0.2 | μM | 32 | Yes | [5, 6] |
|  |  | 0.06-0.5 | μM | 32 | Yes | [5, 6] |
|  |  | 0.35-4.8 | μM | 109 | Yes | [6, 7] |
|  | Conjugated bilirubin fraction in plasma | 0.032 | - | 32 | Yes | [5, 6] |
|  |  | 0.15 | - | 13 | Yes | [8] |
|  | Fecal excretion rate of bilirubin derivatives | 144 | mg/day | 13 | Yes | [9] |
|  | Urinary excretion rate of urobilinogen | 4 | mg/day | -* | Yes | [10] |
|  | Synthesis rate of unconjugated bilirubin | 260 | mg/day | 13 | Yes | [11] |
| Rotor | Unconjugated bilirubin in plasma | 34.2 | μM | 38 | Yes | [12] |
|  |  | 24.79 ± 14.30 | μM | 8 | No | [13] |
|  | Conjugated bilirubin in plasma | 68 ± 57.27 | μM | 38 | Yes | [12] |
|  |  | 53.46 ± 31.44 | μM | 8 | No | [13] |
|  | Total bilirubin in plasma | 78.25 ± 43.96 | μM | 8 | No | [13] |
|  | Conjugated bilirubin fraction in plasma | 67.62 ± 6.78 | μM | 8 | No | [13] |

* Not provided.

**Table S5.** Bilirubin plasma levels in various syndromes.

| **Bilirubin Disorder** | **Observation** | **Concentration [μmol/L]** | **Number of Subjects** | **Reference** |
| --- | --- | --- | --- | --- |
| Gilbert syndrome | Unconjugated bilirubin | 31.5 ± 10.42 | 22 | [5] |
|  | Conjugated bilirubin | 0.29 | 22 | [5, 6] |
|  | Total bilirubin | 32 | 22 | [5, 6] |
| Crigler-Najjar syndrome | Unconjugated bilirubin | 316 | 3 | [5, 6] |
|  | Conjugated bilirubin | 0.7 | 3 | [5, 6] |
|  | Total bilirubin | 316 | 3 | [5, 6] |
| Dubin-Johnson syndrome | Unconjugated bilirubin | 28 | 101 | [6, 14] |
|  | Conjugated bilirubin | 40 | 101 | [6, 14] |
|  | Total bilirubin | 69 | 101 | [6, 14] |

**Table S6.** Comparison of our model with the model by Levitt and Levitt.

|  | **Our Model** | | | | **Levitt and Levitt Model [6]** | | | |
| --- | --- | --- | --- | --- | --- | --- | --- | --- |
|  | **Median Unconjugated Bilirubin [µM]** | **Median Conjugated Bilirubin [µM]** | **Affected Pathway** | **Fractional Reduction** | **Unconjugated Bilirubin [µM]** | **Conjugated Bilirubin [µM]** | **Affected Pathway** | **Fractional Reduction** |
| **Healthy (Reference)** | 4.88 | 0.62 | - | - | 6 | 0.2 | - | - |
| **Rotor Syndrome** | 12.36  (2.5-fold) | 68.84  (111.0-fold) | OATP1B1 | 0 | 34.2  (5.7-fold) | 68.4  (342-fold) |  |  |
| **Gilbert Syndrome** | 29.27  (6.0-fold) | 0.62  (1.0-fold) | UGT1A1 | 0.0075 | 31.5  (5.2-fold) | 0.376  (1.9-fold) | Rate of Glucuronidation | 0.072 |
| **Crigler-Najjar Syndrome** | 326.32  (66.9-fold) | 0.61  (1.0-fold) | UGT1A1 | 0.0009 | 314.6  (53-fold) | 2.34  (11.4-fold) | Rate of Glucuronidation | 0.014 |
| **Dubin-Johnson Syndrome** | 4.83  (1.0-fold) | 39.75  (64.1-fold) | MRP2 | 0.015 | 23.7  (4-fold) | 40  (195-fold) | Biliary Secretion Rate | 0.046 |

**Text S1.** Details of parameter identification and sensitivity analysis.

**Parameter Identification**

Parameters were identified using Monte Carlo algorithm (default settings with log-scaled residuals), implemented in MoBi®. Initially, parameter identification was conducted with all parameters requiring estimation, using equal weights for all observed data (all set to 1). Since this setup underpredicted plasma conjugated bilirubin levels in Rotor syndrome, a second parameter identification was performed in which only the basolateral uptake and efflux rates of unconjugated and conjugated bilirubin were reestimated, assigning a weight of 5 to the conjugated bilirubin data in Rotor syndrome. For all other parameters, the optimal values from the initial identification were used.

In this algorithm, each optimized parameter is individually varied one step upwards and one step downwards around its current value. Subsequently, residuals between observed data and corresponding simulation output are calculated. If the residual sum of squares decreases, the parameter change is accepted. The sequence in which the parameters are varied in each iteration is randomized and each variation step size sampled randomly within the specified parameter ranges. Steps closer to the current parameter value are more likely than larger ones, as determined by projection degree parameter (alpha). If a parameter variation improves the fit, its projection grade is decreased, otherwise it is increased. The process is repeated until the relative error improvement is less than the break condition [15].

The break condition of relative error improvement was set to 0.001. The start value of projection degree (alpha) was 30 and the maximum number of iterations was 10,000. If the optimal value of a parameter was close to upper or lower bound, the current optimal value was used as starting point, and the parameter identification was repeated with an expanded range until the optimal value was not at the boundary.

**Sensitivity Analysis**

Sensitivity analyses were conducted with MoBi®’s default settings. To calculate sensitivity of a pharmacokinetic (PK) parameter of an output (PK_j_) to an input parameter (p_i_) the input parameter was perturbed by a change of Δp_i_, while all other input parameters are unchanged. A new simulation was performed with the perturbed input and the resulting change was computed as the difference between the new and original simulation PK parameter outputs (ΔPK_j_). The sensitivity of PK_j_ to p_i_ was calculated as follows:

$$S_{i, j}=\frac{\Delta PK_{j}}{\Delta p_{i}}\times\frac{p_{i}}{PK_{j}}$$

The resulting sensitivity value is dimensionless. For example, a sensitivity of -1.0 means that increasing the input parameter by 10% causes a 10% reduction in the corresponding PK parameter output. Similarly, a sensitivity of +0.5 indicates that a 10% increase in the input parameter results in a 5% increase of the PK parameter value.

To ensure numerical stability, MoBi® calculates a sensitivity as the average of multiple sensitivity values based on different perturbations Δ_k_ :

$$S_{i, j}=\frac{\sum_{k=1}^{n} \frac{\Delta_{k}PK_{j}}{\Delta_{k}p_{i}}\times\frac{p_{i}}{PK_{j}}}{n}$$

The variation Δ_k_ were defined by multiplication of the original input value with variation factors. The variation factors for each k=1...n are defined as follows:

$$\left( 1+a\times\frac{k}{n} \right) and \left( \frac{1}{1+a\times\frac{k}{n}} \right)$$

where n is number of steps and a is variation range.

We have used the defaults settings in our analyses where number of steps and variation range were 2 and 0.1, respectively. With these values, we obtained four variation factors 1/1.1, 1/1.05, 1.05, and 1.1 [16].

**Text S2.** Partition coefficient and permeability calculation methods.

Organ–plasma partition coefficients were calculated using the PK-Sim Standard method, as implemented in MoBi®. This method extends the approach of Poulin et al. [17, 18] by additionally considering proteins as a tissue component [19]. It is assumed that the unbound concentrations in the aqueous subcompartments of the plasma and tissue are equal at equilibrium and that the whole system is well-stirred [20]. Since only unbound molecules can distribute between compartments, the partition coefficients explicitly incorporate the fraction unbound. Therefore, the low fraction unbound of unconjugated bilirubin is considered in the calculation of partition coefficient.

In the PK-Sim Standard, the partition coefficients are calculated using the following equation [3]:

$$K_{organ}=\left( F_{water}^{organ}+K_{lipid}\times F_{lipid}^{organ}+K_{protein}\times F_{protein}^{organ} \right)\times f_{u}^{plasma}$$

where $F_{water}^{organ}$, $F_{lipid}^{organ}$, and $F_{protein}^{organ}$ are volume fraction of water, lipid, and protein of the organ, respectively. $K_{lipid}$, $K_{protein}$, and $f_{u}^{plasma}$ are lipid/water partition coefficient, protein/water partition coefficient, and fraction unbound in plasma, respectively. Lipophilicity is used for $K_{lipid}$. $K_{protein}$ is calculated from lipophilicity with a correlation determined experimentally measuring the unspecific binding to different tissue protein fraction of various organs for a large set of diverse compounds [21]:

$$K_{protein}= (0.81 + 0.11 \times{10}^{Lipophilicity}) / 24.92 \times5.0$$

Permeability was calculated using the PK-Sim Standard method, as implemented in MoBi®. The permeation rates across the cell membranes are determined by the permeability–surface area (P$\times$SA) product of each organ. The permeability parameters are calculated from the physicochemical properties of the compounds. The ionization of acidic and basic species is not taken into account. It is assumed that this value is constant in all organs and that differences in rate originate only from surface areas. Permeability is calculated as follows [3]:

$$P={(MW_{eff} *{10}^{9} / 336)}^{-6} \times{10}^{Lipophilicity}/ 5 \times{10}^{-4} \times{10}^{-1}$$

where $MW_{eff}$ is effective molecular weight, which is calculated with molecular weights and correction factors for halogen atoms [22].

The low fraction unbound of unconjugated bilirubin is accounted for in the diffusion rate calculations, since the rate is calculated with the unbound fraction of the compounds.

**Text S3.** Data extraction from the Explorys database.

To identify the observations for patients with a healthy bilirubin metabolism, a cohort of patients with polyneuropathy was extracted from the Explorys database, which was henceforth considered as a validation cohort. Individuals with diseases of liver (ICD-10 codes K70-K77), disorders of gallbladder, biliary tract, and pancreas (ICD-10 codes K80-K87), disorders of porphyrin and bilirubin metabolism (ICD-10 code E80), and hemolytic anemias (ICD-10 codes D55-D59) were excluded from the reference cohort. To filter the observations of the patients with the disorders of bilirubin metabolism among liver-disease cohort from the Explorys database, ICD-10 codes of E80.4, E80.5, and E80.6 were used for Gilbert syndrome, Crigler-Najjar syndrome, and both Dubin-Johnson syndrome and Rotor syndrome, respectively. Plasma total, unconjugated, and conjugated bilirubin observations were identified using LOINC codes 1975-2, 1971-1, and 1968-7, respectively. Moreover, observations from patients younger than 18 or older than 65 at the time of measurement were excluded. Furthermore, conjugated bilirubin percentages were calculated if data were available for both conjugated and unconjugated bilirubin, or for total bilirubin along with one of the bilirubin species for the same patient on the same date. Similarly, for subject-specific bilirubin profiles of populations (Figure 5), if data for both conjugated and unconjugated bilirubin were not available but concentration of total bilirubin with one of the bilirubin species was, the concentration of the missing bilirubin species was filled in.

**References:**

1. Tetko, I.V., et al., *Virtual computational chemistry laboratory--design and description.* J Comput Aided Mol Des, 2005. **19**(6): p. 453-63.

2. Levitt, D.G. and M.D. Levitt, *Quantitative assessment of the multiple processes responsible for bilirubin homeostasis in health and disease.* Clin Exp Gastroenterol, 2014. **7**: p. 307-28.

3. Open System Pharmacology Suite. *Compounds: Definition and Work Flows*. 2025 17 September 2025]; Available from: <https://docs.open-systems-pharmacology.org/working-with-pk-sim/pk-sim-documentation/pk-sim-compounds-definition-and-work-flow>.

4. Bourke, E., M.D. Milne, and G.S. Stokes, *Mechanisms of renal excretion of urobilinogen.* Br Med J, 1965. **2**(5477): p. 1510-4.

5. Muraca, M., J. Fevery, and N. Blanckaert, *Relationships between serum bilirubins and production and conjugation of bilirubin: Studies in Gilbert's syndrome, Crigler-Najjar disease, hemolytic disorders, and rat models.* Gastroenterology, 1987. **92**(2): p. 309-317.

6. Levitt, D.G. and M.D. Levitt, *Development of a Pharmacokinetic Model That Accounts for the Plasma Concentrations of Conjugated and Unconjugated Bilirubin Observed in a Variety of Disease States.* Clin Exp Gastroenterol, 2023. **16**: p. 277-289.

7. Doumas, B.T. and T.W. Wu, *The measurement of bilirubin fractions in serum.* Crit Rev Clin Lab Sci, 1991. **28**(5-6): p. 415-45.

8. Berk, P.D., et al., *Studies of bilirubin kinetics in normal adults.* J Clin Invest, 1969. **48**(11): p. 2176-90.

9. Watson, C.J., *Proceedings of the Ninth Congress of the European Society of Haematology.* American Journal of Clinical Pathology, 1963: p. 817.

10. Ha, C. and N.V. Bhagavan, *Hemoglobin and metabolism of iron and heme*. 2023. p. 573-611.

11. Bloomer, J.R., et al., *Comparison of fecal urobilinogen excretion with bilirubin production in normal volunteers and patients with increased bilirubin production.* Clinica Chimica Acta, 1970. **29**(3): p. 463-471.

12. Namihisa, T. and K. Yamaguchi, *The constitutional hyperbilirubinemia in Japan studies on the 139 cases reported during the period from 1963 to 1969.* Gastroenterologia Japonica, 1973. **8**(4): p. 311-321.

13. van de Steeg, E., et al., *Complete OATP1B1 and OATP1B3 deficiency causes human Rotor syndrome by interrupting conjugated bilirubin reuptake into the liver.* J Clin Invest, 2012. **122**(2): p. 519-28.

14. Shani, M., et al., *Dubin-Johnson Syndrome in Israel: I. Clinical, laboratory, and genetic aspects of 101 cases1.* QJM: An International Journal of Medicine, 1970. **39**(4): p. 549-567.

15. Open Systems Pharmacology. *Parameter identification*. 2025 [cited 2025 October 13]; Available from: <https://docs.open-systems-pharmacology.org/shared-tools-and-example-workflows/parameter-identification>.

16. Open System Pharmacology. *Sensitivity analysis*. 2025 [cited 2025 October 13]; Available from: <https://docs.open-systems-pharmacology.org/shared-tools-and-example-workflows/sensitivity-analysis>.

17. Poulin, P., K. Schoenlein, and F.P. Theil, *Prediction of adipose tissue: plasma partition coefficients for structurally unrelated drugs.* Journal of pharmaceutical sciences, 2001. **90**(4): p. 436-447.

18. Poulin, P. and F.P. Theil, *A priori prediction of tissue: plasma partition coefficients of drugs to facilitate the use of physiologically‐based pharmacokinetic models in drug discovery.* Journal of pharmaceutical sciences, 2000. **89**(1): p. 16-35.

19. Kuepfer, L., et al., *Applied Concepts in PBPK Modeling: How to Build a PBPK/PD Model.* CPT: Pharmacometrics & Systems Pharmacology, 2016. **5**(10): p. 516-531.

20. Willmann, S., J. Lippert, and W. Schmitt, *From physicochemistry to absorption and distribution: predictive mechanistic modelling and computational tools.* Expert Opin Drug Metab Toxicol, 2005. **1**(1): p. 159-68.

21. Rouser, G., et al., *Biological membranes*, in *Physical Fact and Function*. 1968, Academic Press New York. p. 5-64.

22. Willmann, S., et al., *A Physiological Model for the Estimation of the Fraction Dose Absorbed in Humans.* Journal of Medicinal Chemistry, 2004. **47**(16): p. 4022-4031.
